# Development of a physiologically-based pharmacokinetic pediatric brain model for prediction of cerebrospinal fluid drug concentrations and the influence of meningitis

**DOI:** 10.1371/journal.pcbi.1007117

**Published:** 2019-06-13

**Authors:** Laurens F. M. Verscheijden, Jan B. Koenderink, Saskia N. de Wildt, Frans G. M. Russel

**Affiliations:** 1 Department of Pharmacology and Toxicology, Radboud University Medical Center, Radboud Institute for Molecular Life Sciences, Nijmegen, The Netherlands; 2 Intensive Care and Department of Pediatric Surgery, Erasmus MC-Sophia Children's Hospital, Rotterdam, The Netherlands; University at Buffalo - The State University of New York, UNITED STATES

## Abstract

Different pediatric physiologically-based pharmacokinetic (PBPK) models have been described incorporating developmental changes that influence plasma drug concentrations. Drug disposition into cerebrospinal fluid (CSF) is also subject to age-related variation and can be further influenced by brain diseases affecting blood-brain barrier integrity, like meningitis. Here, we developed a generic pediatric brain PBPK model to predict CSF concentrations of drugs that undergo passive transfer, including age-appropriate parameters. The model was validated for the analgesics paracetamol, ibuprofen, flurbiprofen and naproxen, and for a pediatric meningitis population by empirical optimization of the blood-brain barrier penetration of the antibiotic meropenem. Plasma and CSF drug concentrations derived from the literature were used to perform visual predictive checks and to calculate ratios between simulated and observed area under the concentration curves (AUCs) in order to evaluate model performance. Model-simulated concentrations were comparable to observed data over a broad age range (3 months–15 years postnatal age) for all drugs investigated. The ratios between observed and simulated AUCs (AUCo/AUCp) were within 2-fold difference both in plasma (range 0.92–1.09) and in CSF (range 0.64–1.23) indicating acceptable model performance. The model was also able to describe disease-mediated changes in neonates and young children (<3m postnatal age) related to meningitis and sepsis (range AUCo/AUCp plasma: 1.64–1.66, range AUCo/AUCp CSF: 1.43–1.73). Our model provides a new computational tool to predict CSF drug concentrations in children with and without meningitis and can be used as a template model for other compounds that passively enter the CNS.

## Author summary

Developmental processes in children affect pharmacokinetics and should ideally be taken into account when establishing drug dosing regimens. One way to incorporate developmental differences is by making use of physiologically-based pharmacokinetic (PBPK) models in which kinetic equations are used to describe drug disposition processes and developmental biology. With these equations the absorption of drugs into the model, the flow of drugs between different compartments (representing major organs/tissues), and excretion from the model are predicted. PBPK models can also be used to describe drug concentrations in different target tissues, which often correlate better with the clinical effects. Here, we developed a generic pediatric PBPK model of drug disposition in the cerebrospinal fluid (CSF), that was able to describe clinically measured drug concentrations of several drugs in neonates and children. The model could be useful in predicting CSF concentrations of other drugs in pediatric populations where clinical data is often sparse or absent and by this means guide first-in-child dose recommendations.

## Introduction

Growth and development significantly impact handling of drugs in children across the pediatric age range. Simple linear bodyweight-based extrapolations from adult to pediatric doses have resulted in toxicity or therapy failure [1]. Taking developmental changes of the processes involved in drug disposition into account in dosing guidelines will lead to improved therapeutic efficacy and safe exposure in children of different ages [2].

Physiologically-based pharmacokinetic (PBPK) modeling is an important tool to simulate drug exposure and design dosing guidelines. PBPK models are compartmental kinetic models in which physiological and drug-specific parameters are as much as possible separated [3]. Physiological parameters describe biological values and processes, and if sufficient data describing developmental biology is available, they can be used to predict plasma drug concentrations in pediatric populations. By this means, PBPK models can guide first-in-child dosing regimens for drugs of which pediatric clinical drug concentrations are scarcely available, resulting in more focused, data-rich clinical trials. There are multiple examples of the successful application of pediatric PBPK models in the drug development process [4].

PBPK models also allow predictions of drug concentrations in target tissues, which often correlate better with the clinical effect. This is especially the case for organs like the brain that are characterized by permeability-limited disposition of various drugs, leading to a significant lag time between their peak plasma and tissue concentrations [5–7]. For drugs acting in the brain, differences in blood and cerebrospinal fluid (CSF) dynamics, blood-brain barrier (BBB) and blood-CSF barrier (BCSFB) permeability, brain and CSF compartment volumes, as well as disease-mediated changes could influence the amount of drug entering the different parts of the central nervous system and should be included to facilitate robust predictions.

Recently, brain PBPK models for adult populations were developed to allow predictions of drug concentrations in brain parenchyma and CSF. For example, Gaohua *et al*. developed an adult brain PBPK model consisting of 4 compartments, which was validated using measured paracetamol CSF concentrations [8]. Yamamoto *et al*. used a different approach by first building a rat model that incorporated multiple brain and CSF compartments [9, 10]. Physiological parameters were in turn adjusted in order to develop a human version [11]. Finally, they extended this adult model into a morphine pediatric brain PBPK model. This model allowed simulations of morphine extracellular fluid concentrations after traumatic brain injury in six children older than 2 years of age [12]. To become more widely accepted, pediatric brain PBPK models should be validated with observed data of different drugs and more individuals across the pediatric age range to increase confidence in the physiological parameters included.

In addition to the validation of a brain PBPK model for a relatively healthy pediatric population, including pathophysiological changes associated with conditions known to affect brain drug concentrations is important to demonstrate model performance in diseased children. Meningitis is a severe condition that leads to impaired BBB and enhanced penetration of drugs into brain and CSF, particularly in newborns and young children [13, 14].

The aim of our study was to describe drug CSF concentrations and describe the effects of meningitis in children by developing and validating a generic pediatric brain PBPK model based on different drugs that enter the brain by passive transfer. Our model provides a new computational tool to predict CSF concentrations in children of drugs that undergo passive transfer and it could serve as a good template for further extension to carrier-mediated transport and disposition in different regions of the central nervous system.

## Results

The full code of the pediatric PBPK model for paracetamol is available in Rstudio format (S1 File). Summary tables of physiological and drug-related parameters are reported in S1 Table and S2 Table, respectively, and can be used to adapt the model to the adult situation and for other drugs. The healthy pediatric brain model was built for paracetamol and subsequently validated using the nonsteroidal anti-inflammatory drugs (NSAIDs) ibuprofen, flurbiprofen and naproxen. BBB permeability of the antibiotic meropenem was optimized in an adult population suffering from meningitis and subsequently simulations were performed for a pediatric population with meningitis and sepsis (methods).

### Building a healthy adult brain PBPK model using paracetamol

**Fig 1:**
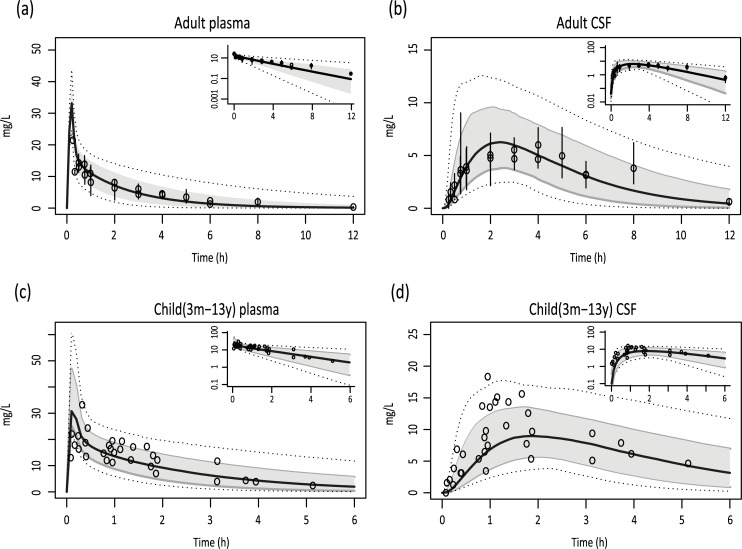
Simulations of paracetamol concentration-time profiles. Plasma and CSF concentration-time profiles of paracetamol after a single intravenous dose of 1000 mg in adults (A, B) and (15 mg/kg) (C,D) in children. Solid black lines indicate simulation of median profiles, the grey area represents 90% CI and dotted lines indicate the minimum and maximum simulation. Dots indicate measured data derived from clinical studies together with the reported S.E.M (adult) or individual observations (pediatric). Log-transformed results are depicted in the right upper corners.

First, the adult brain PBPK model was built and validated using published paracetamol data [7, 15]. Model simulations of plasma and CSF concentrations largely overlaid the observed data and the ratios of the respective AUCs were within twofold difference (Fig 1, Table 1).

**Table 1:** Ratio observed/predicted AUC for plasma and CSF drug concentrations.

Drug	Population	Plasma	CSF
Paracetamol	Adult	1.01	1.18
Paracetamol	Paediatric	1.09	1.23
Ibuprofen (oral)	Paediatric	0.94	1.05
Ibuprofen (IV)	Paediatric	0.92	0.64
Flurbiprofen	Paediatric	0.99	0.76
Naproxen	Paediatric	1.07	0.99
Meropenem	Adult	1.07	1.23
Meropenem	Pediatric(sepsis)	1.66	1.73
Meropenem	Pediatric(meningitis)	1.64	1.41

### Building a healthy pediatric brain PBPK model using paracetamol

**Fig 2:**
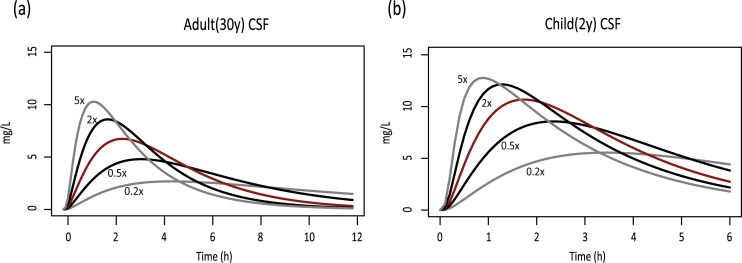
The effect of CSF production rate on paracetamol CSF concentration-time profiles. The red lines indicate the simulations using the default CSF production rate. The black lines indicate a twofold increase or reduction in CSF production rate, respectively. The grey lines indicate a fivefold increase or reduction in CSF production rate, respectively.

After inclusion of the age-related data, simulations of paracetamol concentrations were performed for children aged between 3 months and 13 years. The pediatric model simulations also largely overlaid with observed data (Fig 1). Ratios of simulated over observed plasma and CSF AUCs were also within twofold difference (Table 1). An equivalent dose of approximately 15 mg/kg resulted in a CSF AUC_0-6h_ that was 34% higher in children between 3 months and 13 years of age compared to adults. Because CSF production rate is possibly influenced by co-medication, a sensitivity analysis was performed to investigate the effect of CSF production rate on paracetamol concentration-time profiles [16]. Twofold differences in CSF production had a significant impact on the paracetamol CSF profiles (Fig 2A and 2B), while leaving the shape of the plasma concentration-time curves virtually unaffected (difference in AUC, Cmax and Tmax <1% from original concentration-time profile).

### Validation of the pediatric brain PBPK model using ibuprofen, flurbiprofen and naproxen

**Fig 3:**
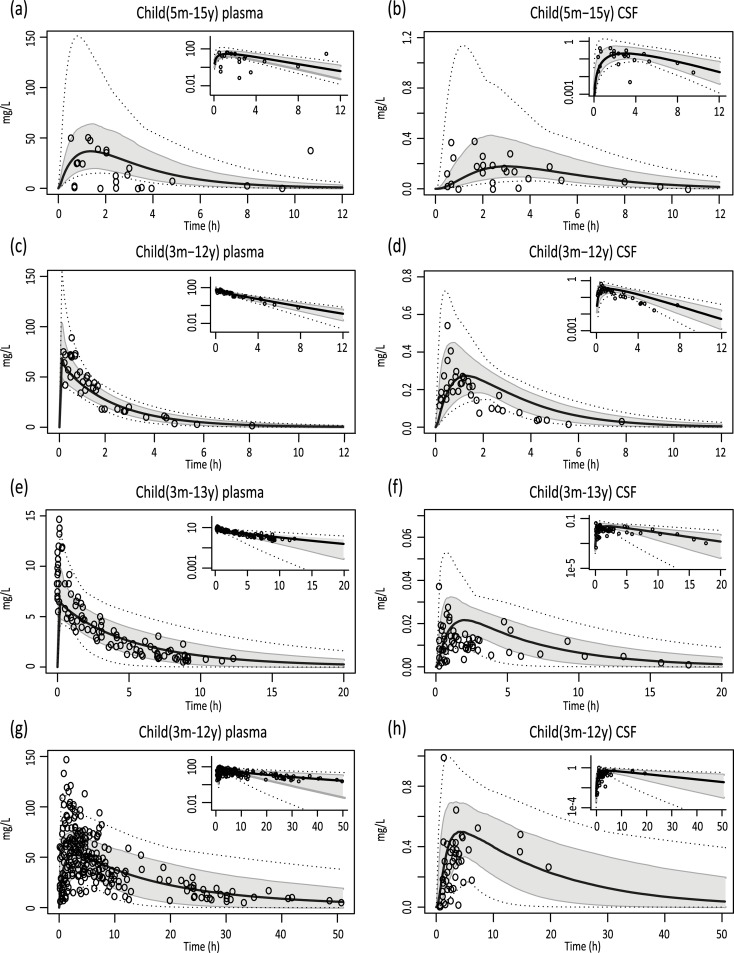
Simulations of pediatric concentration-time profiles for ibuprofen, flurbiprofen and naproxen. Simulations of oral ibuprofen (10 mg/kg in suspected sepsis patients) (A,B), IV ibuprofen (10 mg/kg in surgery patients) (C,D), IV flurbiprofen (0.9 mg/kg in surgery patients) (E,F), and oral naproxen (10 mg/kg in surgery patients) (G,H) concentration-time profiles. Solid black lines indicate simulation of median profiles, the grey area represents 90% CI and dotted lines indicate the minimum and maximum simulation. Dots indicate measured data derived from clinical studies. Log-transformed concentration-time data are depicted in the right upper corners (0 values were discarded).

To validate the physiological parameters included in the paracetamol model for children aged between 3 months and 15 years, simulations were performed with another set of drugs that were not used to build the pediatric model, i.e. ibuprofen, flurbiprofen and naproxen. The model predicted clinically observed data reasonably well, except for flurbiprofen for which plasma volume of distribution seemed to be overestimated. For this drug a Kp scalar of 0.33 (i.e. Kp of every compartment multiplied by 0.33) was introduced to adjust volume of distribution such that simulations of plasma concentrations better correlated with observations (Fig 3). The difference between observed and simulated AUCs for both plasma and CSF were within 2-fold (Table 1).

### Meropenem permeability in the adult brain meningitis PBPK model

**Fig 4:**
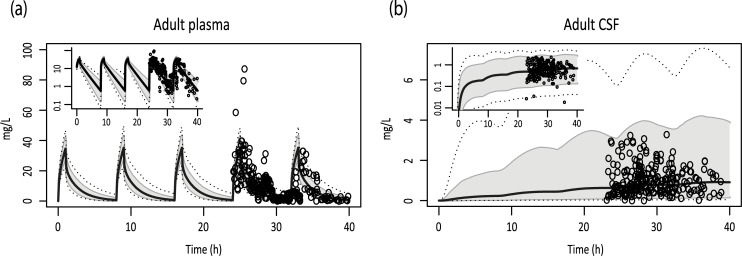
Simulations of adult concentration-time profiles for intravenous meropenem in meningitis patients. Simulations of adult plasma and CSF concentration-time profiles (i.v. 1500 mg/8h) (A,B). Solid black lines indicate simulated median profiles, the grey area represents 90% CI and dotted lines indicate the minimum and maximum simulation. Dots indicate measured data derived from clinical studies. Log-transformed results are depicted in the left upper corners.

Drug-related parameters of meropenem were included in the adult model. No individual doses were reported in the study of Lu *et al*., which was used for validation [17]. Therefore, an intravenous dose of 1500 mg/8h was chosen for our virtual population, which was in the range of the doses used by Lu *et al*. *(*1000 mg/8h, 1000 mg/6h, 2000 mg/8h). Simulations of plasma concentration-time profiles matched well with observed data points. After optimizing permeability in this model, a BBB permeability surface area product of 0.003 L/h (PS 0.0015 L/h for BCSFB) together with a CV of 150% was found (Fig 4, Table 1).

### Meropenem permeability in the pediatric brain meningitis PBPK model

**Fig 5:**
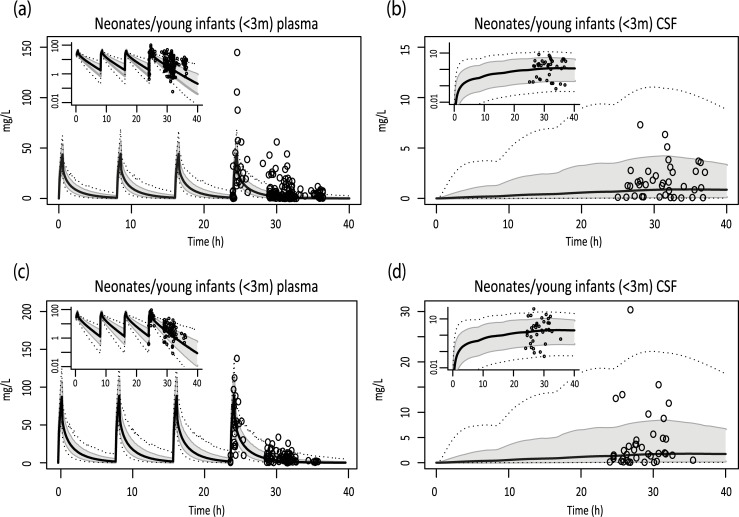
Simulations of pediatric concentration-time profiles for intravenous meropenem. Simulations of plasma and CSF for patients suffering from sepsis (i.v. 20 mg/kg/8h) (A,B), or meningitis (i.v. 40 mg/kg/8h) (C,D). Solid black lines indicate simulated median profiles, the grey area represents 90% CI and dotted lines indicate the minimum and maximum simulation. Dots indicate measured data derived from clinical studies. Log-transformed results are depicted in the left upper corners.

The BBB/BCSFB permeability values estimated in the adult model were also used in the pediatric model for children aged between 1 day and 3 months, after correction for brain weight. This resulted in a <2 fold overlay between pediatric plasma and CSF concentrations both for patients with meningitis and sepsis (Fig 5, Table 1).

## Discussion

The pediatric brain PBPK model described here could predict CSF concentrations of the analgesics paracetamol, ibuprofen and naproxen, and the antibiotic meropenem over a wide age range. The CSF AUCs were simulated within 2-fold error of clinically observed values without the need of changing system parameters of the compartments describing the healthy brain. This shows that parameterization of these compartments was sufficiently robust to allow for simulations of other drugs as well, and that the model could be useful in deriving mechanistically-informed dosing regimens for children.

Pharmacokinetic simulations were validated in relatively healthy children between 3 months and 15 years of age, which resulted in accurate AUC estimates (Table 1). In addition, an attempt was made to perform simulations in children younger than 3 months, including premature neonates, suffering from meningitis/sepsis. Although also in this case the simulated AUCs were within 2-fold of observations, a trend towards an overestimation of plasma and CSF levels could be observed (Table 1). It remains to be elucidated whether this is the result of an influence of age and/or disease, or of the pharmacokinetic simulations in the validation study, which were limited by the availability of only peak and through concentrations.

PBPK models are inherently complex, due to the many different drug-specific and (physiological) system-specific parameters. Their robustness and reliability remain a challenge and there is a clear need to validate model performance with sound experimental data. In this study, most of the physiological processes could be incorporated in an age-appropriate manner, however, due to absence of data, the relative flows between brain compartments expressed as percentage of CSF production rate, were assumed to be the same as in adults.

Our pediatric brain model is structurally similar to that described by Gaohua *et al*. for an adult population [8]. Recently, another pediatric brain PBPK model was developed to simulate concentrations of morphine in brain extracellular fluid of traumatic brain injury patients, but it was only validated with experimental data derived from six children [12]. Although this model provides proof of principle for prediction of brain drug concentrations in children, the pathophysiological changes associated with severe traumatic brain injury may have impacted morphine disposition and hence extrapolation to other patient populations. Other models are based on animal studies and, although they have been validated more extensively, translation to the human situation remains difficult [18, 19].

Depending on the pathophysiological data available, the pediatric model described in this study can be extended with different disease conditions. We simulated drug concentrations in a population of children with meningitis and sepsis. Blood-brain barrier permeability has been described to initially increase during meningitis, although at later times it will return to normal values due to antibiotic-mediated recovery of patients [13]. Since quantitative data on membrane permeability of meropenem were not available from literature, the brain permeability parameter was obtained for the adult population by empirical optimization. The value we derived this way (0.003 L/h) is in the same order of magnitude as intercompartmental clearances that have been described in population PK models for adults and children (0.0017 and 0.0007 L/h, respectively) [17, 20]. This estimated permeability was subsequently applied in simulations with the pediatric model, which is also in accordance with studies in which a correlation between TNFα and blood-brain barrier damage was found, but not between TNFα and age [21].

Simulated and measured maximum concentrations and AUC_csf_/AUC_serum_ ratios were higher in patients suffering from sepsis/meningitis compared to relatively healthy individuals. The highest measured concentration in the healthy population was 1.6 mg/L and the AUC_csf_/AUC_serum_ ratio was 0.047[22]. In our simulations the upper bound of the 90% confidence interval was around 5mg/L and AUC_csf_/AUC_serum_ ratios ranged from 0.09–0.12, indicating that inflammation increased BBB permeability. In the studies of Lu *et al*. and Germovsek *et al*. that we used for validation, samples were taken at different moments after the start of dosing and patients likely differed in disease severity, which might explain the large variability in BBB and BCSFB permeability observed [17, 20]. Time-dependent effects on permeability could, however, not be estimated from the available data. In septic children (without meningitis) the same estimate on meropenem blood-brain barrier permeability resulted in an acceptable overlay between simulations and observed values, which can possibly be explained by a sepsis-induced increase in BBB permeability [23].

In the current model, drugs were included for which carrier-mediated transport does not play a major role in BBB and BCSFB. A next step will be to incorporate membrane transporters in the model for relevant drug substrates. Data on quantitative proteomics of transporter abundance in adults could form the bases for *in vitro-in vivo* extrapolation (IVIVE) of transporter-mediated transport in an adult brain PBPK model [24, 25]. However, for pediatric populations absolute protein expression of BBB/BCSFB transporters has not yet been quantified. Immunohistochemistry studies indicate that expression may not be fully matured in young children as has been described for the ABC transporter P-glycoprotein [26, 27]. Only for very few other transporters information on human ontogeny is available [27].

A limitation of the current study is that data used for validation were obtained in children over a broad age range, which could not be further stratified. Children were suffering from a clinical condition and/or receiving co-medication that could have influenced the pharmacokinetic profiles. Next, CSF drug concentrations were used to validate the simulations and although this is a relevant compartment for antibitiotics during meningitis, the parenchymal extracellular or intracellular fluid is probably more important for other drugs, like analgesics. Future research should be aimed at refining the model by dividing the brain into an intracellular and extracellular space and by expanding the CSF compartments, to better describe the continuum between cranial and spinal CSF. This requires more clinical data on drug disposition in brain tissue and age-appropriate physiological parameters.

It has become more widely accepted that drug research should have an increased focus on pediatric populations to improve safety and reduce off-label dosing [28]. Simple body weight-based scaling ignores developmental processes as illustrated by the simulations in this study with normalized paracetamol doses that did not result in equivalent CSF concentration-time profiles. Modeling and simulation have been recognized as a way to make optimal use of existing data, which could result in more focused clinical trials in children. Moreover, in PBPK modeling, parameters can be partly or completely estimated from *in vitro* or *in silico* studies, further reducing the need for *in vivo* studies [29]. The ultimate goal of pediatric PBPK modeling would be to build models without making use of clinical data and although this might be difficult due to the uncertainty in underlying physiological processes, it could provide guidance for dose selection in first-in-child drug studies. Thereafter, clinical data can be used to improve model performance in a “learn and confirm” cycle [30]. Several published models have shown that it is possible to use a bottom-up *in vitro-in vivo* extrapolation (IVIVE) approach for the estimation of rates of plasma absorption and elimination, which was not yet incorporated in the current model. Linking the brain pediatric PBPK model developed in this study to the existing IVIVE-based models could eventually provide the possibility to mechanistically predict brain concentrations, which would facilitate dosing based on higher quality data as compared to simply scaling from adult dosing regimens.

In conclusion, a mechanistic pediatric PBPK model was developed incorporating 4 different brain compartments, which was used to simulate the plasma and CSF pharmacokinetics of different drugs. The model can be valuable to predict CSF concentrations in cases where clinical data in this compartment is restricted.

## Materials and methods

### Approach

**Fig 6:**
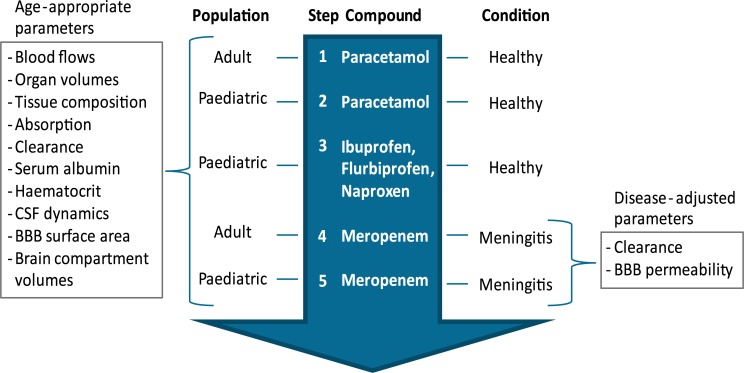
Workflow used for the building and validation of the pediatric brain PBPK model.

A five-step approach was used to build and validate the PBPK brain model for a pediatric population (Fig 6). These steps are briefly summarized below.

Step 1. Building of an adult PBPK brain model (paracetamol)

The adult PBPK brain model developed by Gaohua *et al*. was used as a template and paracetamol as model compound because it is not a substrate for drug transporter proteins expressed in the blood-brain barrier (BBB) or blood CSF barrier (BCSFB) [8].

Step 2. Building of a pediatric PBPK brain model (paracetamol)

Physiological parameters in the adult model were changed to age-appropriate pediatric parameters. Paracetamol was used to allow simulations in relatively healthy children.

Step 3. Validation of the pediatric PBPK brain model (ibuprofen, flurbiprofen, naproxen)

Physiological parameters included in the model were validated further by simulation of plasma and CSF levels of ibuprofen, flurbiprofen and naproxen in relatively healthy children. These drugs were chosen because they are no known substrates for BBB drug transporters, and pediatric CSF concentrations were available in the literature.

Step 4. Building of an adult meningitis PBPK brain model (meropenem)

To the best of our knowledge, no mechanistic data is currently available quantifying the influence of meningitis on brain drug permeability. The impact of meningitis on the passage of meropenem across the BBB and BCSFB was therefore estimated in the adult brain PBPK model (described below step 1) using empirical optimization.

Step 5. Building of a pediatric meningitis PBPK brain model (meropenem)

Meropenem BBB passage estimated in the adult meningitis PBPK model was incorporated in a pediatric model to allow simulations for children with meningitis (combining steps 2, 3 and 4). Because children suffering from sepsis (without meningitis) were also included in the clinical study used for validation, simulations were also done for this population.

At each step, the model was validated using published plasma and CSF concentration data, as described below.

### Step 1 Adult PBPK brain model

#### Plasma model

**Fig 7:**
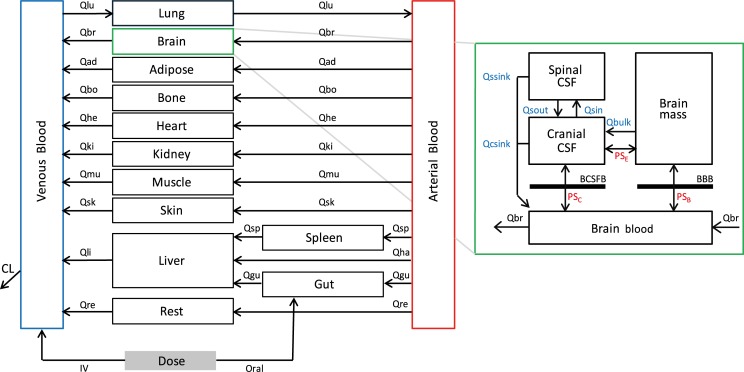
Schematic outline of the PBPK model including four brain compartments (modified from *Gaohua et al*.) [8]. Qsin and Qsout represent CSF shuttle flow between cranial CSF and spinal CSF compartments. Qssink and Qcsink are the flows from CSF compartments to blood. Qbulk represents bulk flow from brain mass to cranial CSF. PS_B_, PS_C_ and PS_E_ represent permeability surface area products between brain blood and brain mass, brain blood and cranial CSF, and brain mass and cranial CSF, respectively. Subscripts lu, br, ad, bo, he, ki, mu, sk, li, re, gu, sp, ha denote lung, brain, adipose tissue, bone, heart, kidney, muscle, skin, liver, rest tissue, gut, spleen and hepatic artery, respectively. CL is the total clearance from the model. IV is an intravenous dose and oral is an oral dose route of administration.

A PBPK model was coded in R software Version 1.1.442 and consisted of 14 compartments representing major organs and tissues (Fig 7). Average physiological parameter values and inter-individual variability were derived from literature [31], or values and equations reported in the Simcyp simulator (Version 17 Release 1) [32–35]. As these are based on weight, height, body surface area, sex and/or age-related equations, correlation between parameters was partly accounted for, because variability in the original parameters is propagated to the estimated/predicted parameter (e.g. a high body weight will on average result in higher organ volumes). Residual parameter variability was assumed to be log-normally distributed. Organ partitioning coefficients were based on previous publications taking into account both logP and ionization of compounds [36–38]. Plasma elimination from the model was included using *in vivo*-measured clearance values reported in literature. Clearance were not extrapolated from *in vitro* experiments, because in this way less robust plasma concentration-time profiles would be generated, which would impede proper assessment of predicted CSF concentrations. Because total body clearance in the adult model was not attributed to specific organs or patient characteristics, only uncorrelated variability in clearance was included. This was described in the adult model as:

$$Pi=Ppop\times e^{Z\eta }$$ (1)

where Pi is the parameter for an individual, Ppop is the population average, Z is the standard normal variable, and η is the variance.

#### Brain model

The brain part of the model was connected to the plasma PBPK model as described before and subdivided into 4 compartments, consisting of brain blood, brain mass, cranial CSF and spinal CSF (Fig 7) [8]. The time-based differential equations used to describe concentration changes in these brain compartments were as follows:

$$V_{bb}*\frac{dCbb}{dt}=Q_{brain}*(C_{bla}-C_{bb})+PS_{b}*(fu_{bm}*C_{bm}-fu_{bb}*C_{bb})+PS_{c}(fu_{ccsf}*C_{ccsf}-fu_{bb}*C_{bb})+Q_{ssink}*C_{scsf}+Q_{csink}*C_{ccsf}$$ (2)

$$V_{bm}\frac{dCbm}{dt}=PS_{b}*(fu_{bb}*C_{bb}-fu_{bm}*C_{bm})+PS_{e}*(fu_{ccsf}*C_{ccsf}-fu_{bm}*C_{bm})-Q_{bulk}*C_{bm}$$ (3)

$$V_{ccsf}*\frac{dC_{ccsf}}{dt}=PS_{e}*(fu_{bm}*C_{bm}-fu_{ccsf}*C_{ccsf})+PS_{c}*(fu_{bb}*C_{bb}-fu_{ccsf}*C_{ccsf})+Q_{bulk}*C_{bm}+Q_{sout}*C_{scsf}-Q_{sin}*C_{ccsf}-Q_{csink}*C_{ccsf}$$ (4)

$$V_{scsf}*\frac{dC_{scsf}}{dt}=Q_{sin}*C_{ccsf}-Q_{sout}*C_{scsf}-Q_{ssink}*C_{scsf}$$ (5)

Where *V*_*bb*_, *V*_*bm*_, *V*_*ccsf*_, *V*_*scsf*_, *fu*_*bm*_, *fu*_*bb*_, *fu*_*ccsf*_, *fu*_*scsf*_, *C*_*bb*_, *C*_*bm*_, *C*_*ccsf*_ and *C*_*scsf*_ represent volumes, unbound fractions and concentrations in brain blood, brain mass, cranial CSF and spinal CSF, respectively. *Q*_*brain*_ denotes brain blood flow and *C*_*bla*_ denotes concentration in arterial blood. *Q*_*sin*_ and *Q*_*sout*_ represent CSF shuttle flow between cranial CSF and spinal CSF compartments. *Q*_*ssink*_ and *Q*_*csink*_ are the flows from CSF compartments to blood. *Q*_*bulk*_ represents bulk flow from brain mass to cranial CSF. *PS*_*b*_, *PS*_*c*_ and *PS*_*e*_ represent permeability surface area products between brain blood and brain mass, brain blood and cranial CSF, and brain mass and cranial CSF, respectively.

The following assumptions were made: (1) The BBB is a barrier between brain mass and brain blood, and the BCSFB between cranial CSF and brain blood. The barrier separating brain mass and cranial CSF is of high permeability. No barrier exists between cranial CSF and spinal CSF. (2) Compartments are of constant volume and well-stirred. (3) Permeability surface area products of the BCSFB are two-fold smaller than corresponding BBB values, as was previously described [8, 11, 39]. (4) As drug metabolism was included as a total-body clearance, a specific contribution of brain metabolism is assumed absent and drug entering the brain mass compartment is returned into brain blood to preserve mass balance. (5) Transporter-mediated transfer across barriers is considered negligible for the studied drugs, as they are no known substrates for drug transporters expressed in BBB or BCSFB. Therefore, barrier penetration is considered to occur by passive diffusion and described by permeability surface area products [24].

(6) CSF production rate was multiplied by 2 in populations receiving spinal ketamine anesthesia, based on a two-fold increase that was observed in cats [16]. (7) A brain tissue binding parameter was derived from literature or estimated using the prediction option incorporated in Simcyp [32–35].

BBB permeability was estimated for each drug separately from rat carotid artery perfusion data or cell-based passive permeability assays, and in the latter case scaled to *in vivo* values by using the equation:

PSbbb=invitropermeability×BBBsurface (6)

where *in vitro* permeability is expressed as dm/h, and BBB surface in dm^2^. A sensitivity analysis was performed to investigate the effect of CSF production rate change on the drug concentration-time curve in the spinal CSF compartment.

### Step 2 Pediatric PBPK brain model

#### Plasma model

To translate the adult model to a pediatric model estimates of height, weight, body surface area, organ volumes, tissue blood flows, hematocrit and albumin concentrations were adjusted using previously reported equations from literature [31] or the pediatric Simcyp simulator [32–34]. This resulted in an age-appropriate set of parameters for each simulated patient. Child-specific tissue composition was also incorporated, which affects the prediction of organ partitioning coefficients. Pediatric plasma clearances were not estimated by the model, but derived from literature (S2 Table, partially also from the same studies used for validation of the brain PBPK model) and consisted of body weight-based relations. The age range for the patients in these studies overlapped with that of the simulated patients almost completely, to reduce unjustified extrapolation of clearance values across ages. For oral dosing studies a rate constant (ka) and variability was derived from literature, and if this value was unavailable an estimate was made by using the MechPeff model incorporated in Simcyp [32–34]. Unexplained variability for clearance and absorption was incorporated using Eq 1 (previous section).

#### Brain model

The time-based differential equations and assumptions made in the adult brain PBPK model were also applied to the pediatric model. In addition, physiological values for brain parameters were derived from literature leading to the following considerations: (1) Brain volume, brain blood flow, spinal CSF volume and CSF production rate were adjusted as a function of age [31, 35, 40–43]. (2) Cranial CSF volume was not expected to further increase after birth [35, 44]. (3) Relative CSF flows between compartments, expressed as percentage of CSF production, were assumed to be similar to adult values. (4) Since BBB surface area per gram of brain is similar for adults and children, total surface area was smaller in children because of a lower brain mass compared to adults [31, 43, 45] (Table 2).

**Table 2:** Pediatric brain physiological parameters.

Volumes(L)^a^			
**Equation**	**Description**	**Notes**	**Ref.**
$V_{brain\;total}=10*\frac{\frac{age+0.315}{9+6.92*age}}{1.04}$	Brain volume		[31]
*V*_*brain blood*_ = 0.05 * *V*_*brain*_	Brain blood volume		[8, 35]
*V*_*ccsf*_ = 0.143	Cranial CSF volume		[35, 44]
$V_{scsf}=\frac{1.94*body\;weight+0.13}{1000}$ $Limit\;V_{scsf}\leq \frac{0.143}{0.8}*0.2$	Spinal CSF volume	Spinal CSF volume capped at 20% of total CSF volume (same as in adult)	[35, 40]
*V*_*endothelial*_ = *V*_*brain total*_ * 0.005	Endothelial cell volume		[35]
*V*_*brain mass*_ = *V*_*brain total*_−*V*_*endothelial*_−*V*_*brain blood*_−*V*_*ccsf*_−*V*_*scsf*_	Brain mass volume		[35]
**Fluid flow rates(L/h)**			
**Equation**	**Description**	**Notes**	**Ref.**
*Q*_*csfproductionrate*(3*m*−18*y*)_ = 0.024 $Q_{csfproductionrate(<3m)}=\frac{4.007*\log\left(age\right)+7.088}{1000}$	CSF production rate	If ketamine used in clinical study. Production rate multiplied by 2.	[8, 35, 41, 42]
*Q*_*csfproductionrate* (*CV*%)_ = 10			
*Q*_*bulk*_ = 0.25*Q_*csfproductionrate*_	Bulk flow brain mass to cranial CSF	Relative CSF flows (as part of Q_csfproductionrate_) assumed to be the same for adults and children.	[8, 35]
*Q*_*bulk* (*CV*%)_ = 8			[8, 35]
*Q*_*sin*_ = *Q*_*ssink*_+*Q*_*sout*_	Flow from cranial CSF to spinal CSF		[8, 35]
*Q*_*sout*_ = 0.9*Q_*ssink*_	Flow from spinal CSF to cranial CSF		[8, 35]
*Q*_*sout* (*CV*%)_ = 100			[8, 35]
*Q*_*csink*_ = 0.75 * *Q*_*csfproductionrate*_+*Q*_*bulk*_−*Q*_*sin*_+*Q*_*sout*_	Flow from cranial CSF to brain blood		[8, 35]
*Q*_*ssink*_ = 0.38*(0.75*Q_*csfproductionrate*_+*Q*_*bulk*_)	Flow from spinal CSF to brain blood		[8, 35]
*Q*_*ssink* (*CV*%)_ = 30			[8, 35]
$Q_{brain}=Qcarout*\frac{10+2290*{(10}^{-0.608*age}-10^{-0.639*age})}{100}$	Brain blood flow	Cardiac output * fractional tissue flow	[35]
**BBB surface area (m^2^)**			
$BBB=BBB_{adult}*\frac{V_{\mathrm{brain}\;\mathrm{total}\;child}}{V_{\mathrm{brain}\;\mathrm{total}\;adult}}$	BBB surface area		[31, 43, 45]

^a^ Tissue volumes were converted to liters. Adult organ densities reported in Abduljalil *et al*. [46] were used to convert equations predicting organ weight to organ volumes if needed.

### Step 3 Validation of pediatric brain PBPK model

To validate the physiological parameters included in the pediatric PBPK model, plasma and CSF concentrations were simulated with different drugs not used to build the model, namely ibuprofen, flurbiprofen, and naproxen using both adult and pediatric models. Only drug-specific parameters were adjusted to perform simulations for these drugs, of which brain penetration is like paracetamol known not to be affected by drug transporters.

### Step 4 Incorporation of meningitis in the adult model

In case of meningitis, blood-brain barrier function is known to be impaired [47]. To have an estimate on permeability of meropenem through the BBB the permeability surface area product was empirically optimized in the adult meropenem model, both for the population average and coefficient of variation. Effects of meningitis on plasma clearance was already incorporated in the plasma parameter derived from the study that was also used for validation of the model [17]. In addition, effects of meningitis on free fraction and erythrocyte-plasma partitioning coefficients were not incorporated, because differences in albumin concentrations and hematocrit levels were assumed to be of minor importance due to low protein binding and low cell penetration of meropenem [48–51].

### Step 5 Incorporation of meningitis in the pediatric model

The value for blood-brain barrier permeability estimated in the adult model was also used in the pediatric model after correction for the difference in blood-brain barrier surface area. The effect of meningitis on plasma clearance was already incorporated in the clearance parameter derived from the NeoMero study, in which only patients were included suffering from sepsis or meningitis [20]. Effects of meningitis on free fraction and erythrocyte-plasma partitioning coefficients were not incorporated, as also for the pediatric population this was assumed to be of minor importance (see previous section).

### In vivo observations and model evaluation

Clinical studies describing concentration-time profiles of paracetamol in adults (step 1) and paracetamol (step 2), ibuprofen, flurbiprofen and naproxen (step 3), in children, were used for validation and extracted from original publications with WebPlotDigitizer version 4.1 [7, 15, 17, 20, 52–56]. As plasma and CSF samples were taken in the setting of clinical care, the majority of data was derived from individuals having a clinical condition, however, except for patients suffering from sepsis and/or meningitis, this was not expected to affect brain permeability (Table 3). Studies included healthy adult volunteers (1 study [15]), adult nerve root compression pain/arthritis patients (1 study [7]), pediatric surgery patients receiving spinal anesthesia (4 studies [52–55]) and pediatric (suspected) sepsis patients (1 study [56]). For step 4 (adults) and step 5 (children) meropenem PK studies, all patients suffered from (suspected) sepsis and/or meningitis (2 studies [17, 20]). In the study of *Germovsek et al*. concentrations were described as ‘time after dose’ at steady state, which was expected to be reached after 24h [57]. Because in this study meropenem CSF values for children suffering from sepsis (without meningitis) were available, simulations were performed for this population as well.

**Table 3:** Characteristics of studies included for validation.

Study	Drug	Number of patients	Co-medication	Age(y)	Indication	CSF collection	Percentage male (%)
Singla et al., 2012 [15]	Paracetamol	7	-	19–44	Healthy	Spinal catheter	100
Bannwarth et al., 1992 [7]	Propacetamol (similar to 50% dose of paracetamol) [58]	43	-	31–73	Nerve-root compression pain	Diagnostic lumbar puncture	56
Kumpulainen et al., 2007 [52]	Paracetamol	32	Midazolam, ketamine, propofol, thiopental	0.25–13	Elective surgery	Lumbar puncture for spinal anesthesia	59
Välitalo et al., 2012 [53]	Naproxen	53	Midazolam, ketamine, propofol, thiopental	0.25–12	Surgery lower part body	Lumbar puncture for spinal anesthesia	74
Kumpulainen et al., 2010 [54]	Flurbiprofen	27	Midazolam, ketamine, propofol, thiopental, paracetamol, ketoprofen, fentanyl	0.25–13	Surgery lower part body	Lumbar puncture for spinal anesthesia	78
Kokki et al., 2007 [55]	Ibuprofen	36	Midazolam, ketamine	0.25–12	Surgery	Lumbar puncture for spinal anesthesia	69
Har-Even et al., 2014 [56]	Ibuprofen	28	-	0.42–15	Suspected sepsis	Lumbar puncture for sepsis assessment	64
Lu et al., 2016 [17]	Meropenem	82	- not reported	17–77	Meningitis	Lumbar drainage	61
Germovsek et al., 2018 [20]	Meropenem	167	- not reported	0.0027–0.25	Sepsis/ Meningitis	Opportunistic lumbar puncture for sepsis/meningitis assessment	53

Simulations were run for 1000 virtual individuals who were matched with the individuals in the original studies for dosing regimen, age range, and percentage male/female. Simulations of CSF concentration-time profiles were visualized for the spinal compartment as clinical measurements were performed by lumbar puncture. Results were compared with observations using visual predictive checks in which median, 5^th^ percentile, 95^th^ percentile, minimum, and maximum values were overlaid with clinical observations derived from literature. Also, plasma and CSF AUC_0-last_ were calculated for median simulated data and for observed data using a non-compartmental (linear trapezoidal) approach. A naïve pooling approach was used for the population data because variability between subjects was expected to cancel out in the analysis. In case of two observations at the same time, the average was used. AUCs were compared between simulated data and observed data according to:

$$fold\;error=\frac{observed\;AUC}{predicted\;AUC}$$ (7)

and model simulations were considered acceptable if the ratio was within two-fold difference [59]. Only plasma peak and through levels were available for the pediatric population receiving meropenem and few high CSF values largely influenced AUC. This means that requirements for the use of the naïve pooling approach are not met [60]. In contrast, AUC was for this study calculated below the simulated concentration time profile reported.

## Supporting information

S1 File Rstudio code. (R)

S1 Table Physiological parameters. (PDF)

S2 Table Drug-related parameters. (PDF)
