# Supplementary material for: Development of a physiologically-based pharmacokinetic pediatric brain model for prediction of cerebrospinal fluid drug concentrations and the influence of meningitis
Source: PLoS Comput Biol. 2019 Jun 13;15(6):e1007117. doi: 10.1371/journal.pcbi.1007117 (PMC6592555; doi:10.1371/journal.pcbi.1007117)
Supplement: S1 Table — (PDF) [file pcbi.1007117.s002.pdf]

## S1 Table Physiological parameters

| | | | | |
|---|---|---|---|---|
| ***Volumes(L)***[a] | | | | |
| | **Pediatric** | **Adult** | **notes** | **Ref.** |
| Vbrain total | (10*(age+0.315)/(9+6.92*age))/1.04 | (1.449 – 3.62/BW)/1.04 | | [1] |
| Vbrain blood | 0.05*Vbraintotal | 0.05*Vbraintotal | | [2] |
| Vccsf | 0.143 | Male:<br>Vbrainmale*0.105*0.8<br>Female:<br>Vbrainfemale*0.092*0.8 | | [2, 3] |
| Vscsf | (1.94*BW+0.13)/1000<br>Limit Vscsf <= (0.143/0.8)*0.2 | Male:<br>Vbrainmale*0.105*0.2<br>Female:<br>Vbrainfemale*0.092*0.2 | Pediatric Vscsf volume capped at 20% of total csf (20% same in adult) | [2, 4] |
| Vendothelial mass | Vbrainmale*0.005 | Vbrainmale*0.005 | | [2] |
| Vbrain mass | Vbrain-Vbrainendo-Vbb-Vccsf-Vscsf | Vbrain-Vbrainendo-Vbb-Vccsf-Vscsf | | [2] |
| Vlung | Male: (29.08*(Height/100) * Weight^0.5 +11.06 + 35.47*(Height/100) * Weight^0.5 +5.53)/1000)/1.05<br>Female:<br>((31.46*(Height/100) * Weight^0.5 + 1.43 + 35.3*(Height/100) * Weight^0.5 +1.53)/1000)/1.05 | Male:<br>(29.08*(Height/100) * Weight^0.5 +11.06 + 35.47*(Height/100) * Weight^0.5 +5.53)/1000)/1.05<br>Female:<br>((31.46*(Height/100) * Weight^0.5 + 1.43 + 35.3*(Height/100) * Weight^0.5 +1.53)/1000)/1.05 | | [1] |
| Vadipose | Age<1:<br>((908.4 + 0.706 * (Weight*1000) - 53*Height + 358.5*gender - 3.057 * (age*365))/1000)/0.92<br>Age 1_3 male:<br>((908.4 + 0.706*(Weight*1000) - 53*Height + 358.5*0-3.057*(1.1*365))/1000)/0.92<br>Age 3-11 male:<br>(0.534*Weight - 1.59*age + 3.03) /0.92<br>Age >12 male:<br>(1.36*Weight)/(Height/100) – 42<br>Age 1_3 female:<br>((908.4 + 0.706*(Weight*1000) - 53*Height + 358.5*1 - 3.057*(1.1*365))/1000)/0.92<br>Age 3_12 female:<br>(0.642*Weight - 0.12*Height-0.606*age+ 8.98)/0.92<br>Age >12 female:<br>(1.61*Weight)/(Height/100) - 38.3 | Male:<br>(1.36*Weight)/(Height/100) – 42<br>Female:<br>(1.61*Weight)/(Height/100) - 38.3 | | [1] |
| Vbone | Age 0_1:<br>(((77.24 + 24.94*Weight + 0.21 * | Male:<br>((Weight – | | [1] |

| | | | | |
|---|---|---|---|---|
| | (age *365) - 1.889 * Height)/0.33)/1000)/1.3<br>Age 1_3:<br>(((77.24 + 24.94*Weight + 0.21 * (1.1*365) - 1.889 * Height) /0.33)/1000)/1.3<br>Age 3_18:<br>(((7.4*Height + 28.9*Weight - 789.6)/0.4)/1000)/1.3 | Vadipose*0.92 )/1.3*0.058)<br><br>Female:<br>((Weight-Vadipose*0.92)/1.3*0.051) | | |
| Vheart | Male:<br>((22.81*(Height/100)*Weight^0.5 - 4.15)/1000)/1.05<br>Female:<br>((19.99*(Height/100)*Weight^0.5 - 1.53)/1000)/1.05 | Male:<br>(155.18*(BSA)^1.29)/1000/1.05<br>Female:<br>(124.13*(BSA)^1.242)/1000/1.05 | | [1] |
| Vkidney | (4.214*Weight^0.823 + 4.456*Weight^0.795)/1000 | ((15.4+2.04*Weight+51.8*(Height/100)^2)/1000)/1.05 | | [1] |
| Vmuscle | Male:<br>(0.3 + ((0.54-0.3)/18)*age)*(Weight-Vadipose*0.92)/1.04<br>Female:<br>0.3 + ((0.489-0.3)/18)*age)*(Weight-Vadipose*0.92)/1.04 | Male:<br>(0.244*Weight + 7.8*(Height/100)-0.098*age+3.3)/1.04<br>Female:<br>(0.244*Weight + 7.8*(Height/100)-0.098*age-3.3)/1.04 | | [1] |
| Vskin | (BSA/1000)*45.655+(BSA/1000)*1240 | (BSA/1000)*45.655+(BSA/1000)*1240 | Skin thickness was derived from values in simcyp simulator (adult) | [1, 2] |
| Vspleen | Male:<br>((8.74*(Height /100) * Weight ^0.5+11.06)/1000)*1.06<br>Female:<br>((9.36*(Height/100)*Weight^0.5+7.98)/1000)/1.06 | (6.516*Weight^0.797)/1000 | | [1] |
| Vgut | Male:<br>(0.021* (Weight-Vadipose*0.92))/1.05<br>Female:<br>(0.021* (Weight-Vadipose*0.92))/1.05 | Male:<br>0.021* (Weight-Vadipose*0.92)/1.05<br>Female:<br>0.027* (Weight-Vadipose*0.92)/1.05 | | [1] |
| Vliver | Male:<br>((576.9*(Height/100) + 8.9*Weight - 159.7)/1000)/1.05<br>Female:<br>((674.3*(Height/100) + 6.5*Weight - 214.4)/1000)/1.05 | (1072.8*(BSA) - 345.7)/1000 | | [1] |
| Vbla | Age<1:<br>((10^(0.7891*(LOG10(Weight)+0.004132*Height+1.8117)))/1000)/2<br>Age 2_14 male:<br>((10^(0.6459*(LOG10(Weight))+0.002743*(Height)+2.0324))/1000)/2<br>Age 2_6 female: | Male:<br>(((13.1*Height+ 18.05*Weight - 480)/0.5723)/1000)/2<br>Female:<br>(((35.5*Height + 2.27*Weight - | | [1] |

| | | | | |
|---|---|---|---|---|
| | ((10^(0.6459*(LOG10(Weight))+0.002743*(Height)+2.0324))/1000)/2<br>Age 7_14:<br>((10^(0.6412*(LOG10(Weight))+0.00127*(Height)+2.2169))/1000)/2 | 3382)/0.6178)/1000)/2 | | |
| Vblv | Age<1:<br>((10^(0.7891*(LOG10(Weight)+0.004132*Height+1.8117)))/1000)/2<br>Age 2_14 male:<br>((10^(0.6459*(LOG10(Weight))+0.002743*(Height)+2.0324))/1000)/2<br>Age 2_6 female:<br>((10^(0.6459*(LOG10(Weight))+0.002743*(Height)+2.0324))/1000)/2<br>Age 7_14:<br>((10^(0.6412*(LOG10(Weight))+0.00127*(Height)+2.2169))/1000)/2 | Male:<br>(((13.1*Height+ 18.05*Weight - 480)/0.5723)/1000)/2<br>Female:<br>(((35.5*Height + 2.27*Weight - 3382)/0.6178)/1000)/2 | | [1] |
| Vrest | Weight-Vblv-Vbla-Vliver-Vgut-Vspleen-Vskin-Vmuscle-Vkidney-Vheart-Vbone-Vadipose-Vlung-Vbrain | Weight-Vblv-Vbla-Vliver-Vgut-Vspleen-Vskin-Vmuscle-Vkidney-Vheart-Vbone-Vadipose-Vlung-Vbrain | Body density of 1 kg/L assumed | - |

| ***Fluid flow rates(L/h)*** | | | | |
|---|---|---|---|---|
| | **Pediatric** | **Adult** | | |
| Qproductionrate | 3m-18y:0.024<br><3m:(4.007*log10(age)+7.088)/1000 | 0.021 | If ketamine used in clinical study. Production rate multiplied by 2. | [2, 5-7] |
| Qproductionrate (CV) | 10% | 10% | | |
| Qsin | Q_ssink+Q_sout | Q_ssink+Q_sout | Assumed: relative CSF flows (as part of Qcsfproductionrate) same for adults and children. | [2, 6] |
| Qsout | 0.9*Qssink | 0.9*Qssink | | [2, 6] |
| Qsout (CV) | 100% | 100% | | |
| Qcsink | 0.75*Q_productionrate+Q_bulk-Qsin+Q_sout | 0.75*Q_productionrate +Q_bulk-Qsin+Q_sout | | [2, 6] |
| Qssink | 0.38*(0.75*Qproductionrate+Qbulk) | 0.38*(0.75*Qproductionrate+Qbulk) | | [2, 6] |
| Qssink (CV) | 30% | 30% | | |
| Qbulk | 0.25*Qproductionratemale | 0.25*Qproductionrate male | | [2, 6] |
| Qbulk (CV) | 8% | 8% | | |
| Qbrain | Qcarout* (( 10 + 2290 *(EXP(-0.608*age) - EXP(-0.639*age)))/100) | Qcarout*0.12 | Cardiac output * fractional tissue flow | [2] |
| Qcarout | BSA* (110 + (184.974 * (EXP(-0.0378 * age) - EXP(-0.2477 * age)))) | BSA * 60 * (3 - 0.01 * (age- 20)) | | [2] |
| Qlung | Qcarout*1 | Qcarout*1 | Cardiac output * fractional tissue flow | [2] |
| Qadipose | <u>Male:</u><br>Qcarout*0.05<br><u>Female:</u><br>Qcarout*(( 5 + (3.59 * age^5/(14.49^5 + age^5)))/100) | <u>Male:</u><br>Qcarout*0.05<br><u>Female:</u><br>Qcarout*0.085 | Cardiac output * fractional tissue flow | [2] |
| Qbone | Qcarout*0.05 | Qcarout*0.05 | Cardiac output * fractional tissue flow | [2] |
| Qheart | <u>Male:</u><br>Qcarout*0.04<br><u>Female:</u><br>Qcarout*0.05 | <u>Male:</u><br>Qcarout*0.04<br><u>Female:</u><br>Qcarout*0.05 | Cardiac output * fractional tissue flow | [2] |
| Qkidney | <u>Male:</u><br>Qcarout* ((4.53 + (14.63*age^1/(0.188^1 + age ^1)))/100)<br><u>Female:</u><br>Qcarout*((4.53 + (13*age^1.15/(0.188^1.15 + age^1.15)))/100) | <u>Male:</u><br>Qcarout*0.19<br><u>Female:</u><br>Qcarout*0.17 | Cardiac output * fractional tissue flow | [2] |
| Qmuscle | <u>Male:</u><br>Qcarout* ((6.03 + (12*age^2.5/(11^2.5 + | <u>Male:</u><br>Qcarout*0.17<br><u>Female:</u> | Cardiac output * fractional | [2] |

| | | | | |
|---|---|---|---|---|
| | age^2.5)))/100)<br>Female:<br>Qcarout* ((6.03 + (7*age^2.5/(12^2.5 + age^2.5)))/100) | Qcarout*0.12 | tissue flow | |
| Qskin | Qcarout* ((1.0335 + (4*age^5/ (5.16^5 + age^5)))/100) | Qcarout*0.05 | Cardiac output * fractional tissue flow | [2] |
| Qspleen | Male:<br>Qcarout*0.02<br>Female:<br>Qcarout*0.03 | Male:<br>Qcarout*0.02<br>Female:<br>Qcarout*0.03 | Cardiac output * fractional tissue flow | [2] |
| Qgut | Male:<br>Qcarout*0.15<br>Female:<br>Qcarout*0.17 | Male:<br>Qcarout*0.15<br>Female:<br>Qcarout*0.17 | Cardiac output * fractional tissue flow | [2] |
| Qha | Qcarout*0.065 | Qcarout*0.065 | Cardiac output * fractional tissue flow | [2] |
| Qliver | Male:<br>Qcaroutmale[i]*0.235<br>Female:<br>Qcaroutfemale[i]*0.265 | Male:<br>Qcarout*0.235<br>Female:<br>Qcarout*0.265 | Cardiac output * fractional tissue flow | [2] |
| Qrestflow | Qcarout-Qbrain-Qadipose-Qbone-Qheart-Qkidney-Qmuscle-Qskin-Qspleen-Qgut-Qha | Male:<br>Qcarout*0.095<br>Female:<br>Qcarout*0.09 | Qcarout minus organ flows | - |

| Tissue composition (fraction) | | | | |
|---|---|---|---|---|
| Adipose | FractionEWadipose (32.154 - 2.7863 * log10(age))*(14.1/18/100)<br>FractionIWadipose= (32.154 - 2.7863 * log10(age))*(3.9/18/100)<br>FractionNLadipose= (35.5 + 43.46 * age/(1.5 + age))*(79/79.2/100)<br>FractionNPadipose= (35.5 + 43.46 * age/(1.5 + age))*(0.2/79.2/100) | EW=0.141<br>IW=0.039<br>NL=0.79<br>NP=0.002 | EW=extracellular water<br>IW=intracellular water<br>NL=neutral lipids<br>NP=neutral phospholipids | [2] |
| Bone | FractionEWbone=(64.179 - 1.2697 * age)*(9.8/43.9/100)<br>FractionIWbone=(64.179 - 1.2697 * age)*(34.1/43.9/100)<br>FractionNLbone= (0.2 + 0.3655 * age)*(7.4/7.51/100)<br>FractionNPbone= (0.2 + 0.3655 * age)*(0.11/7.51/100) | EW=0.098<br>IW=0.341<br>NL=0.074<br>NP=0.0011 | | [2] |
| Gut | FractionEWgut= (75.378 - 0.3932 * log10(age))*(26.7/71.8/100)<br>FractionIWgut= (75.378 - 0.3932 * log10(age))*(45.1/71.8/100)<br>FractionNLgut= (2.5 + 0.185 * age)*(4.87/6.5/100)<br>FractionNPgut= (2.5 + 0.185 * age)*(1.63/6.5/100) | EW=0.267<br>IW=0.451<br>NL=0.0487<br>NP=0.0163 | | [2] |
| Heart | FractionEWheart =(84.523 - 0.4249 * age)*(31.3/75.8/100)<br>FractionIWheart= (84.523 - 0.4249 * age)*(44.5/75.8/100)<br>FractionNLheart= (2.3159 + 0.0797 * age)*(1.15/2.81/100)<br>FractionNPheart= (2.3159 + 0.0797 * age)*(1.66/2.81/100) | EW=0.313<br>IW=0.445<br>NL=0.0115<br>NP=0.0166 | | [2] |
| Kidney | FractionEWkidney= (83.278 - 0.2162 * age)*(28.3/78.3/100)<br>FractionIWkidney= (83.278 - 0.2162 * age)*(50/78.3/100)<br>FractionNLkidney= (2.73 + 1.995 * age /(2.59 + age))*(2.07/3.69/100)<br>FractionNPkidney= (2.73 + 1.995 * age /(2.59 + age))*(1.62/3.69/100) | EW=0.283<br>IW=0.50<br>NL=0.0207<br>NP=0.0162 | | [2] |
| liver | FractionEWliver= (75.69 - 0.573 * log10(age))*(16.5/75.1/100)<br>FractionIWliver= (75.69 - 0.573 * log10(age))*(58.6/75.1/100)<br>FractionNLliver= (3 + 3.089 * age/(1.8 + age))*(3.48/6/100)<br>FractionNPliver= (3 + 3.089 * age/(1.8 + age))*(2.52/6/100) | EW=0.165<br>IW=0.586<br>NL=0.0348<br>NP=0.0252 | | [2] |
| Lung | FractionEWlung= (80.973 - 0.4916 * log10(age))*(34.8/81.1/100)<br>FractionIWlung= (80.973 - 0.4916 * log10(age))*(46.3/81.1/100)<br>FractionNLlung= (1.857 - 0.211 * log10(age))*(0.3/1.2/100)<br>FractionNPlung= (1.857 - 0.211 * log10(age))*(0.9/1.2/100) | EW=0.348<br>IW=0.463<br>NL=0.003<br>NP=0.009 | | [2] |
| Muscle | FractionEWmuscle= (77.211 - | EW=0.091 | | [2] |

| | | | | |
|---|---|---|---|---|
| | 0.4321 * log10(age))*(9.1/76/100)<br>FractionIWmuscle= (77.211 - 0.4321 * log10(age))*(66.9/76/100)<br>FractionNLmuscle= (1.9852 + 0.0649 * age)*(2.38/3.1/100)<br>FractionNPmuscle= (1.9852 + 0.0649 * age)*(0.72/3.1/100) | IW=0.667<br>NL=0.0238<br>NP=0.0072 | | |
| Skin | FractionEWskin= (72.395 - 1.1462 * log10(age))*(62.3/71.77/100)<br>FractionIWskin= (72.395 - 1.1462 * log10(age))*(9.47/71.77/100)<br>FractionNLskin=3.95*(2.84/3.95/100)<br>FractionNPskin=3.95*(1.11/3.95/100) | EW=0.623<br>IW=0.0947<br>NL=0.0284<br>NP=0.0111 | | [2] |
| Spleen | FractionEWspleen= (79.952 - 0.4178 * log10(age))*(20.8/78.7/100)<br>FractionIWspleen= (79.952 - 0.4178 * log10(age))*(57.9/78.7/100)<br>FractionNLspleen=(1.5 + 0.015 * age)*(2.01/3.99/100)<br>FractionNPspleen=(1.5 + 0.015 * age)*(1.98/3.99/100) | EW=0.208<br>IW=0.579<br>NL=0.0201<br>NP=0.0198 | | [2] |
| plasma | FractionNLplasma= (0.5578 + 0.036 * log10(age))*(0.35/0.57/100)<br>FractionNPplasma= (0.5578 + 0.036 * log10(age))*(0.22/0.57/100) | EW=0.945<br>IW=0<br>NL=0.0035<br>NP0.0022 | | [2] |

| | | | | |
|---|---|---|---|---|
| **Rest** | | | | |
| Hematocrit | Male:<br>(53 - ((43.0 * age^1.12 / (0.05^1.12 + age^1.12)) * (1 + (-0.93 * age^0.25 / (0.10^0.25 + age^0.25)))))/100<br>Female:<br>(53 - ((37.4 * age^1.12 / (0.05^1.12 + age^1.12)) * (1 + (-0.80 * age^0.25 / (0.10^0.25 + age^0.25)))))/100 | Male:<br>0.43<br>Female:<br>0.38 | | [2] |
| HSA (g/L) | 33.746 + 1.1287 * log10(365*age) | 45 | | [2] |
| Height | Male:<br>0.0000176179*age^7 - 0.00119874*age^6 + 0.0323848*age^5 - 0.444112*age^4 + 3.2946*age^3 - 13.2191*age^2 + 33.75*age + 52.62152<br>Female:<br>-0.00000151027*age^8 + 0.000121261*age^7 - 0.0040023*age^6 + 0.070179*age^5 - 0.708233*age^4 + 4.1872*age^3 -14.3393*age^2 + 33.84778*age + 51.535477 | Male:<br>175.32 + 0.1113 * age-0.0025 * age^2<br>Female:<br>161.66 + 0.1319 * age-0.0027 * age^2 | | [2] |
| Height (CV) | Male:<br>5.7<br>Female:<br>5.2 | Male:<br>3.9<br>Female:<br>3.9 | Coefficient of variation (%) | [2] |
| Weight | Male:<br>7.826 * (1 - EXP(age * -1.2)) + EXP((Height * 0.0209) + (0.023 * age))<br>Female:<br>5.454 * (1 - EXP(age* -1.57)) + EXP((Height* 0.0224) + (0.019 * age)) | Male:<br>EXP(2.643 + 0.0099 *Height)<br>Female:<br>EXP(2.7383 + 0.0091 * Height) | | [2] |
| Weight (CV) | Male:<br>15<br>Female:<br>10 | Male:<br>15<br>Female:<br>18.8 | Coefficient of variation (%) | [2] |
| BSA | 0.007184*Height^0.725*Weight^0.425 | 0.007184*Height^0.725*Weight^0.425 | | [2] |

[a]Tissue volumes were converted to liters. Adult organ densities reported in Abduljalil[8] were used to convert equations predicting organ weight to organ volumes if needed.

1. Price PS, Conolly RB, Chaisson CF, Gross EA, Young JS, Mathis ET et al. Modeling interindividual variation in physiological factors used in PBPK models of humans. Crit Rev Toxicol. 2003;33(5):469-503.
2. Simcyp. 17.0.90.0 ed. available from: https://www.certara.com/.
3. Andescavage NN, DuPlessis A, McCarter R, Vezina G, Robertson R, Limperopoulos C. Cerebrospinal Fluid and Parenchymal Brain Development and Growth in the Healthy Fetus. Dev Neurosci. 2016;38(6):420-9. doi:10.1159/000456711.
4. Rochette A, Malenfant Rancourt MP, Sola C, Prodhomme O, Saguintaah M, Schaub R et al. Cerebrospinal fluid volume in neonates undergoing spinal anaesthesia: a descriptive magnetic resonance imaging study. Br J Anaesth. 2016;117(2):214-9. doi:10.1093/bja/aew185.
5. Blomquist HK, Sundin S, Ekstedt J. Cerebrospinal fluid hydrodynamic studies in children. J Neurol Neurosurg Psychiatry. 1986;49(5):536-48.
6. Gaohua L, Neuhoff S, Johnson TN, Rostami-Hodjegan A, Jamei M. Development of a permeability-limited model of the human brain and cerebrospinal fluid (CSF) to integrate known physiological and biological knowledge: Estimating time varying CSF drug concentrations and their variability using in vitro data. Drug Metab Pharmacokinet. 2016;31(3):224-33. doi:10.1016/j.dmpk.2016.03.005.
7. Yasuda T, Tomita T, McLone DG, Donovan M. Measurement of cerebrospinal fluid output through external ventricular drainage in one hundred infants and children: correlation with cerebrospinal fluid production. Pediatr Neurosurg. 2002;36(1):22-8. doi:10.1159/000048344.
8. Abduljalil K, Jamei M, Johnson TN. Fetal Physiologically Based Pharmacokinetic Models: Systems Information on the Growth and Composition of Fetal Organs. Clin Pharmacokinet. 2018. doi:10.1007/s40262-018-0685-y.
