# Supplementary material for: Development of a physiologically-based pharmacokinetic pediatric brain model for prediction of cerebrospinal fluid drug concentrations and the influence of meningitis
Source: PLoS Comput Biol. 2019 Jun 13;15(6):e1007117. doi: 10.1371/journal.pcbi.1007117 (PMC6592555; doi:10.1371/journal.pcbi.1007117)
Supplement: S2 Table — (PDF) [file pcbi.1007117.s003.pdf]

S2 Table Drug related parameters

Paracetamol

Parameter	Value		Units	Notes	References
	Adult	Pediatric			
PSb (BBB)	1.875	Vbrain/(1.36/1.04) *1.875	L/h	Adult brain weight assumed 1.36kg[1]	[2]
PSe (brain-CCSF)	300	300	L/h	Assumed to be no barrier	
PSc(BCSFB)	0.9375	0.5*Vbrain/(1.36/1.04)*1.875	L/h	PSc =1/2*PSb	
Fubm(mass)	0.747	0.747			[3]
Fupl	0.855	0.855			[3]
Fuccsf	1	1		Assumed (low conc. protein in CSF)	
Fuscsf	1	1		Assumed (low conc. protein in CSF)	
BP	1.09	1.09		1-hematocrit+ EP*hematocrit	
CLiv	22.8	17.6*(Weight/70)^(1.204-0.454*Weight^1.4/(12.2^1.4+Weight^1.4))	L/h		[4, 5]
	0.354	0.354		variance	[5]
Mol weight	151.2	151.2	g/mol		[6]
logP	0.46	0.46			[6]
Type compound	Monoprotic acid	Monoprotic acid			
pKa	9.38	9.38			[6]
EP	1.2	1.2			[6]

Ibuprofen

Parameter	Value	Units	Notes	References
	Pediatric			
PSb (BBB)	0.0947*Vbrain*1.04*1000	L/h		[7]
PSe (brain-CCSF)	300	L/h	Assumed to be no barrier	
PSc(BCSFB)	0.5*0.0947*Vbrain*1.04*1000	L/h	PSc =1/2*PSb	
Fubm(mass)	0.3117			[8]
Fupl	(0.00567+0.0107)/2			[9, 10]
Fuccsf	1		Low amount protein in CSF	
Fuscsf	1		Low amount protein in CSF	
BP	1		1-hematocrit+ EP*hematocrit	
CLiv	0.0738*0.965*Weight	L/h		[11]
	0.09		variance	[11]
Ka	1.08	h-1		Mechpeff model simcyp simulator V17
Ka	30	%		Mechpeff model simcyp simulator V17
Fraction absorbed	0.965			[12]
Mol weight	206.285	g/mol		[13]
logP	3.97			[13]
Type compound	Monoprotic acid			
pKa	4.91			[13]
EP	1		When no EP was reported a value of 1 was used	

Flurbiprofen

Parameter	Value	Units	Notes	References
	Pediatric			
PSb (BBB)	0.0567*Vbrain*1.04*1000	L/h		[7]
PSe (brain-CCSF)	300	L/h	Assumed to be no barrier	
PSc(BCSFB)	0.5*0.0567*Vbrain*1.04*1000	L/h	PSc =1/2*PSb	
Fubm(mass)	0.257		Simcyp predicted value	
Fupl	0.005			[14-16]
Fuccsf	1		Low amount protein in CSF	
Fuscsf	1		Low amount protein in CSF	
BP	1		1-hematocrit+ EP*hematocrit	
CLiv	0.96*(Weight/70)^0.5	L/h		[17]
	0.28		variance	[17]
Mol weight	244.265	g/mol		[18]
logP	4.16			[18]
Type compound	Monoprotic acid			
pKa	4.42			[19]
EP	1		When no EP was reported a value of 1 was used	

Naproxen

Parameter	Value	Units	Notes	References
	Pediatric			
PSb (BBB)	26.4*Vbrain/(1.36/1.04)	L/h	Adult brain weight assumed 1.36kg[1]. Adult BBB assumed 15m^2[6].	[20]
PSe (brain-CCSF)	300	L/h	Assumed to be no barrier	
PSc(BCSFB)	0.5*26.4*Vbrain*(1.36/ 1.04)	L/h	PSc =1/2*PSb	
Fubm(mass)	0.497			[21]
Fupl	0.01			[22]
Fuccsf	1		Low amount protein in CSF	
Fuscsf	1		Low amount protein in CSF	
BP	1		1-hematocrit+ EP*hematocrit	
CLiv	0.62*(weight/70)*Fabs	L/h		[23]
CLiv	0.49		variance	[23]
Ka	1.1	h-1		[23]
	0.77		variance	[23]
Fraction absorbed	1			[22]
Mol weight	230.263	g/mol		[24]
logP	3.18			[24]
Type compound	Monoprotic acid			
pKa	4.15			[24]
EP	1		When no EP was reported a value of 1 was used	

Meropenem

Parameter	Value		Units	Notes	references
	Adult	Pediatric			
PSb (BBB)	0.003	0.5*Vbrain/(1.36/1.04)*0.003	L/h	Adult brain weight assumed 1.36kg[1]	Scaled value
	150	150	%	Coefficient of variation	Scaled value
PSe (brain-CCSF)	300	300	L/h	Assumed to be no barrier	
PSc(BCSFB)	0.5*0.003	0.5*0.003*Vbrain/(1.36 /1.04)	L/h	PSc =1/2*PSb	
	150%	150%		Coefficient of variation	
Fubm(mass)	0.999	0.999		Simcyp predicted value	
Fupl	0.79	0.79			[25]
Fuccsf	1	1		Low amount protein in CSF	
Fuscsf	1	1		Low amount protein in CSF	
BP	0.69	0.69		1-hematocrit+ EP*hematocrit	
CLiv	22.2	16.7*(Weight/70)	L/h		[26, 27]
	0.224	0.255		variance	[26, 27]
Mol weight	383.463	383.463	g/mol		[28]
logP	-0.6	-0.6			[28]
Type compound	ampholyte	ampholyte			
pKa	3.47; 9.39	3.47; 9.39			[29]
EP	0.3	0.3		Assumed based on alveolar cell blood ratio	[30]

1. Dekaban AS. Changes in brain weights during the span of human life: relation of brain weights to body heights and body weights. Ann Neurol. 1978;4(4):345-56. doi:10.1002/ana.410040410.
2. Summerfield SG, Dong KC. In vitro, in vivo and in silico models of drug distribution into the brain. J Pharmacokinet Pharmacodyn. 2013;40(3):301-14. doi:10.1007/s10928-013-9303-7.
3. Summerfield SG, Lucas AJ, Porter RA, Jeffrey P, Gunn RN, Read KR et al. Toward an improved prediction of human in vivo brain penetration. Xenobiotica. 2008;38(12):1518-35. doi:10.1080/00498250802499459.
4. Prescott LF. Kinetics and metabolism of paracetamol and phenacetin. Br J Clin Pharmacol. 1980;10 Suppl 2:291S-8S.
5. Wang C, Allegaert K, Tibboel D, Danhof M, van der Marel CD, Mathot RA et al. Population pharmacokinetics of paracetamol across the human age-range from (pre)term neonates, infants, children to adults. J Clin Pharmacol. 2014;54(6):619-29. doi:10.1002/jcph.259.
6. Gaohua L, Neuhoff S, Johnson TN, Rostami-Hodjegan A, Jamei M. Development of a permeability-limited model of the human brain and cerebrospinal fluid (CSF) to integrate known physiological and biological knowledge: Estimating time varying CSF drug concentrations and their variability using in vitro data. Drug Metab Pharmacokinet. 2016;31(3):224-33. doi:10.1016/j.dmpk.2016.03.005.
7. Parepally JM, Mandula H, Smith QR. Brain uptake of nonsteroidal anti-inflammatory drugs: ibuprofen, flurbiprofen, and indomethacin. Pharm Res. 2006;23(5):873-81. doi:10.1007/s11095-006-9905-5.
8. Longhi R, Corbioli S, Fontana S, Vinco F, Braggio S, Helmdach L et al. Brain tissue binding of drugs: evaluation and validation of solid supported porcine brain membrane vesicles (TRANSIL) as a novel high-throughput method. Drug Metab Dispos. 2011;39(2):312-21. doi:10.1124/dmd.110.036095.
9. Lockwood GF, Albert KS, Szpunar GJ, Wagner JG. Pharmacokinetics of ibuprofen in man--III: Plasma protein binding. J Pharmacokinet Biopharm. 1983;11(5):469-82.
10. Ochs HR, Greenblatt DJ, Verburg-Ochs B. Ibuprofen kinetics in patients with renal insufficiency who are receiving maintenance hemodialysis. Arthritis Rheum. 1985;28(12):1430-4.
11. Gelotte CK, Prior MJ, Pendley C, Zimmerman B, Lavins BJ. Multiple-dose pharmacokinetics and safety of an ibuprofen-pseudoephedrine cold suspension in children. Clin Pediatr (Phila). 2010;49(7):678-85. doi:10.1177/0009922810363153.
12. Atkinson HC, Stanescu I, Frampton C, Salem, II, Beasley CP, Robson R. Pharmacokinetics and Bioavailability of a Fixed-Dose Combination of Ibuprofen and Paracetamol after Intravenous and Oral Administration. Clin Drug Investig. 2015;35(10):625-32. doi:10.1007/s40261-015-0320-8.
13. Pubchem. Ibuprofen. https://pubchem.ncbi.nlm.nih.gov/compound/3672. Accessed 18-12-2018.
14. Evrard PA, Cumps J, Verbeeck RK. Concentration-dependent plasma protein binding of flurbiprofen in the rat: an in vivo microdialysis study. Pharm Res. 1996;13(1):18-22.
15. Risdall PC, Adams SS, Crampton EL, Marchant B. The disposition and metabolism of flurbiprofen in several species including man. Xenobiotica. 1978;8(11):691-703.
16. Wanwimolruk S, Birkett DJ, Brooks PM. Protein binding of some non-steroidal anti-inflammatory drugs in rheumatoid arthritis. Clin Pharmacokinet. 1982;7(1):85-92.
17. Kumpulainen E, Valitalo P, Kokki M, Lehtonen M, Hooker A, Ranta VP et al. Plasma and cerebrospinal fluid pharmacokinetics of flurbiprofen in children. Br J Clin Pharmacol. 2010;70(4):557-66. doi:10.1111/j.1365-2125.2010.03720.x.
18. Pubchem. Flurbiprofen. https://pubchem.ncbi.nlm.nih.gov/compound/3394. Accessed 18-12-2018.
19. Drugbank. Flurbiprofen. https://www.drugbank.ca/drugs/DB00712. Accessed 18-12-2018.

20. Corti G, Maestrelli F, Cirri M, Mura P, Zerrouk N. Dissolution and permeation properties of naproxen from solid-state systems with chitosan. Drug Deliv. 2008;15(5):303-12. doi:10.1080/10717540802006955.
21. Cheng Z, Zhang J, Liu H, Li Y, Zhao Y, Yang E. Central nervous system penetration for small molecule therapeutic agents does not increase in multiple sclerosis- and Alzheimer's disease-related animal models despite reported blood-brain barrier disruption. Drug Metab Dispos. 2010;38(8):1355-61. doi:10.1124/dmd.110.033324.
22. Davies NM, Anderson KE. Clinical pharmacokinetics of naproxen. Clin Pharmacokinet. 1997;32(4):268-93. doi:10.2165/00003088-199732040-00002.
23. Valitalo P, Kumpulainen E, Manner M, Kokki M, Lehtonen M, Hooker AC et al. Plasma and cerebrospinal fluid pharmacokinetics of naproxen in children. J Clin Pharmacol. 2012;52(10):1516-26. doi:10.1177/0091270011418658.
24. CID=156391 NCfBIPCD. https://pubchem.ncbi.nlm.nih.gov/compound/156391 (accessed Jan. 31, 2019).
25. Isla A, Maynar J, Sanchez-Izquierdo JA, Gascon AR, Arzuaga A, Corral E et al. Meropenem and continuous renal replacement therapy: in vitro permeability of 2 continuous renal replacement therapy membranes and influence of patient renal function on the pharmacokinetics in critically ill patients. J Clin Pharmacol. 2005;45(11):1294-304. doi:10.1177/0091270005280583.
26. Lu C, Zhang Y, Chen M, Zhong P, Chen Y, Yu J et al. Population Pharmacokinetics and Dosing Regimen Optimization of Meropenem in Cerebrospinal Fluid and Plasma in Patients with Meningitis after Neurosurgery. Antimicrob Agents Chemother. 2016;60(11):6619-25. doi:10.1128/AAC.00997-16.
27. Germovsek E, Lutsar I, Kipper K, Karlsson MO, Planche T, Chazallon C et al. Plasma and CSF pharmacokinetics of meropenem in neonates and young infants: results from the NeoMero studies. J Antimicrob Chemother. 2018. doi:10.1093/jac/dky128.
28. Pubchem. Meropenem. https://pubchem.ncbi.nlm.nih.gov/compound/441130. Accessed 18-12-2018.
29. Drugbank. Meropenem. https://www.drugbank.ca/drugs/DB00760. Accessed 18-12-2018.
30. Nicolau DP. Pharmacokinetic and pharmacodynamic properties of meropenem. Clin Infect Dis. 2008;47 Suppl 1:S32-40. doi:10.1086/590064.
